# Water, sanitation, and hygiene access in southern Syria: analysis of survey data and recommendations for response

**DOI:** 10.1186/s13031-018-0151-3

**Published:** 2018-04-23

**Authors:** Mustafa Sikder, Umar Daraz, Daniele Lantagne, Roberto Saltori

**Affiliations:** 1 Department of Civil and Environmental Engineering, Tufts University, Medford, MA USA; 2 Whole of Syria Water, Sanitation, and Hygiene Sector, UNICEF MENARO (Middle East and North Africa Regional Office), Amman, Jordan

**Keywords:** Emergency response, Water supply, Hygiene, Sanitation, Syria, Water safety plan

## Abstract

**Background:**

Water, sanitation, and hygiene (WASH) are immediate priorities for human survival and dignity in emergencies. In 2010, > 90% of Syrians had access to improved drinking water. In 2011, armed conflict began and currently 12 million people need WASH services. We analyzed data collected in southern Syria to identify effective WASH response activities for this context.

**Methods:**

Cross-sectional household surveys were conducted in 2016 and 2017 in 17 sub-districts of two governorates in opposition controlled southern Syria. During the survey, household water was tested for free chlorine residual (FCR). Descriptive statistics were calculated, and mixed effect logistic regressions were completed to determine associations between demographic and WASH variables with outcomes of FCR > 0.1 mg/L in household water and reported diarrhea in children < 5 years old.

**Results:**

In 2016 and 2017, 1281 and 1360 surveys were conducted. Piped water as the main water source declined from 22.0% to 15.3% over this time. Households accessed 50–60 l per capita daily (primarily from private water trucking networks). Households spent ~ 20% of income on water and reported market-available hygiene items were unaffordable. FCR > 0.1 mg/L increased from 4.1% to 27.9% over this time, with Water Safety Plan (WSP) programming strongly associated with FCR (mOR: 24.16; 95% CI: 5.93–98.5). The proportion of households with childhood diarrhea declined from 32.8% to 20.4% over this time; sanitation and hygiene access were protective against childhood diarrhea.

**Conclusions:**

The private sector has effectively replaced decaying infrastructure in Syria, although at high cost and uncertain quality. Allowing market forces to manage WASH services and quantity, and targeting emergency response activities on increasing affordability with well-targeted subsidies and improving water quality and regulation via WSPs can be an effective, scalable, and cost-effective strategy to guarantee water and sanitation access in protracted emergencies with local markets.

## Background

Emergencies, including natural disasters, disease outbreaks, and complex emergencies, are occurring at increasing rates, and affecting an increasing number of people [1–4]. As classified by the United Nations, *complex emergencies* are defined as ‘situations of disrupted livelihoods and threats to life produced by warfare, civil disturbance, and large-scale movements of people, in which any emergency response has to be conducted in a difficult political security environment [5]. Today, more than 1.5 billion people are threatened by conflict and violence [6]; and there are more than 65 million displaced persons worldwide, the highest number ever recorded [7].

Water, sanitation, and hygiene (WASH) are immediate priorities for human survival and dignity in emergencies [8]. WASH interventions commonly implemented in emergency response in households and facilities include: 1) water supply, including construction or repair of water infrastructure, and support for operation and maintenance of systems; 2) water treatment, including operationalizing simple water safety plans at central and household levels; 3) excreta disposal, supporting sewage systems or constructing emergency facilities; and, 4) promotion of hygiene practices and provision of hygiene items [8]. WASH interventions in middle income or urban settings generally focus on supporting existing large-scale infrastructure, while interventions for displaced populations generally focus on facility construction. Adequate WASH coverage in emergencies may prevent displacement, reduce risk of outbreaks, reduce risk of malnutrition, and play a fundamental role in dignity, protection, school attendance, and livelihoods [9]. WASH interventions reduce both the risk of disease and the risk of transmission of disease [10], although program design, implementation characteristics, and community aspects were found critical to program success in a recent systematic review.

In March 2011, armed conflict began in Syria. Today, 13.5 million Syrians are estimated in need of humanitarian assistance [11], 6.1 million are internally displaced within Syria [12], and an estimated 12 million need WASH services [13]. Before the conflict, Syria provided > 90% of its population with access to improved drinking water, according to the definitions and indicators then used to track the Millennium Development Goals [14]. Because of conflict, infrastructure functionality progressively deteriorated, mainly due to lack of power supply [15]. Unavailability of spare parts and consumables due to international sanctions and insecurity, displacement of trained professionals, lack of investment in preventive maintenance, and lack of financial resources also contributed to decreased utility functionality. Additionally, water has been used as weapon of war, by all parties in the conflict. In some cases, portions of the civil population have been deprived of centralized supply for protracted periods of time.

Since August 2015, Security Council resolution 2165 authorized the United Nations to carry out humanitarian operation inside Syria, in areas under the control of different actors, with cross border operations. A coordination mechanism termed the Whole of Syria (WoS) was established, with the goals of coordinating interventions from different hubs and maximizing humanitarian action efficiency. The WoS 2017 Humanitarian Response Plan focuses on three objectives: saving lives, ensuring protection, and increasing resilience and access to services [16]. The WASH Cluster is responsible for coordinating WASH responses to meet these objectives, and has collected data on WASH access within Syria to inform response efforts. As Syria is an example of an emerging type of complex emergency (a protracted conflict in a middle-income context with pre-existing infrastructure and vibrant local market), data collection is particularly relevant as traditional intervention strategies are less than optimal. Currently, the WASH sector in Syria is: 1) providing support to urban infrastructure and promoting Water Safety Plans (WSPs) as a risk-based community management strategy; and, 2) promoting traditional WASH activities of small-scale emergency infrastructure, hygiene kit provision, and community mobilization.

Water Safety Plans (WSP) are a systematic approach that consistently ensures the safety of drinking-water supply through the use of a comprehensive risk assessment and risk management approach encompassing all steps in the water supply from catchment to consumer [17]. Depending on the situation, WSPs vary in complexity. In case of southern Syria, WSP implementation involved conducing a risk assessment at three levels (household, trucked water system, and piped network system) followed by implementation appropriate risk management measures. The implemented risk management measures included chlorination training, distribution of chlorine and chlorine testing equipment, installation of chlorination stations at the water collection wells, household- and community- based water safety awareness campaigns, and fixing water lines and pumps of the supply network.

Our goal was to analyze WoS WASH sector data collected in opposition controlled southern Syria to identify the most effective WASH interventions.

## Methods

**Fig. 1:**
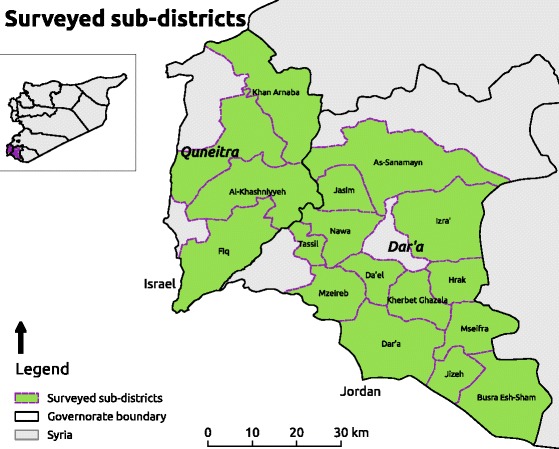
Geographic location of the surveyed sub-districts in the two governorates of southern Syria

In June/July 2016 and February 2017, under the umbrella of the Whole of Syria WASH cluster, the working group in the Amman hub (comprising of UNICEF as sector lead and other program implementation partners), conducted a household survey to assess WASH services in 17 sub-districts of Dar’a (13 sub-district comprised of 50 communities) and Quneitra (4 sub-districts comprised of 14 communities) governorates where Amman hub operates humanitarian assistance (Fig. 1). The sampling frame was households in opposition-controlled areas, where safe access was possible. The data were collected at the same time by all implementing partners.

A list of sub-districts and their population was prepared using the Humanitarian Needs Overview (HNO) 2015 data [13]. Sample size was calculated using the Krejcie and Morgan model [18]; set for 95% confidence with 10% margin of error and one degree of freedom, to allow for generalizable results by sub-district. The calculated sample size was 87–96 households per sub-district; this was increased to 106 to account for response rate. The number of households sampled in communities within sub-districts was proportionate to population. The individual household randomization process within a community varied, but followed a systematic method (e.g. if maps were available the sample was divided equally by neighborhood or random routes were used). Permanent residents and internally displaced persons (IDPs) of urban and rural areas were surveyed. Enumerators were trained on ethical survey administration; local community councils were informed, and household consent was obtained before conducting the survey. If armed conflict prevented access to a community, data collection was deferred until the area became accessible. Secondary analysis of de-identified data was approved by the Institutional Review Board of Tufts University (IRB Study #1706006).

### Household survey

A 24-question survey was administered by trained enumerators in the Syrian Arabic language to gather information on household demographics, water collection, storage, and consumption practices, sanitation, and waste management status, hygiene behaviors of household members, and self-reported diarrhea over the last two weeks. The questionnaire survey was conducted electronically through Open Data Kit (University of Washington, Seattle, WA, USA). Please note the survey tool is available upon request from the corresponding author.

### Water quality testing

During the survey, drinking water samples from households were collected by the enumerator. The enumerator tested FCR (range 0.1–3.0 mg/L) and pH (range 6.8–8.2) using a Lovibond Colorimeter (The Tintometer Ltd., Amesbury, UK), DPD-1 tablets, and phenol red solution.

### Statistical analysis

Data were saved in Microsoft Excel (Microsoft Corp., Redmond, WA, USA) format from the server, and cleaned and analyzed using R 3.3.3 (R Foundation for Statistical Computing, Vienna, Austria). First, households with no children under five years of age were excluded from the dataset. Then, initial data analysis was performed by creating, tabulating, and graphing individual demographic and WASH variables. T-tests were used to assess statistically significant differences between 2016 and 2017 data at the *p* < 0.05 level. Then, forward selection stepwise logistic regression was completed. We first compared demographic and WASH variables in single variate analysis against the outcomes of FCR > 0.1 mg/L and reported diarrhea in children < 5 years old. Variables where the 95% confidence interval of unadjusted odds ratios (uOR) did not spanning the null value (uOR = 1) were included in logistic regressions. Secondly, statistically associated (*p*-value< 0.05) variables from the logistic model were including in a mixed effect regression to address the variability introduced by sub-districts.

## Results

### Household survey

The survey was conducted in 17 sub-districts in southern Syria (Fig. 1). In 2016 and 2017, 1825 and 1921 complete responses were obtained, with a mean of 107.4 (range 105–116) and 101.2 (range 74–120) households per sub-district in 2016 and 2017, respectively. Of these, 70.2% (2016) and 70.8% (2017) of households had at least one child under five.

### 2016 Household WASH assessment

#### Demographics

In 2016, 971 responses from Dar’a and 310 responses from Quneitra were obtained (Table 1). The average household size was 7.6 persons, with 31.1% of respondents IDPs. Overall, 19.4% of households were headed by women, and average head of household age was 42.3 years. Just under half of respondents (46.5%) reported living in damaged or shared shelters.

**Table 1:** Descriptive statistics from 2016 survey results

	Governorate [min and max per sub district]		Total
	Dar’a	Quneitra	
HH has at least one child < 5 years, % (n)	69.4% (971) [50.5–78.3%]	73.0% (310) [67.9–77.4%]	70.2% (1281)
Mean (SD) people per household	7.5 (3.6) [6.1–8.9]	8.1 (4.3) [7.2–9.2]	7.6 (3.8)
IDP households, % (n)	25.4% (247) [1.9–44.6%]	49.0% (152) [29.6–85.4%]	31.1% (399)
Female headed HH, % (n)	18.9% (184) [5.7–47.0%]	21.0% (65) [17.3–23.3%]	19.4% (249)
Mean (SD) age of the head of HH	41.8 (12.8) [34.2–45.7]	44.1 (12.3) [42.4–45.3]	42.3 (12.7)
Families in damaged/shared shelters, % (n)	44.9% (436) [16.7–81.1%]	51.6% (160) [32.1–78.1%]	46.5% (596)
Water trucking main source in last 30 days, % (n)	73.0% (709) [36.1–100%]	89.3% (277) [81.3–97.5%]	77.0% (986)
Network main source in last 30 days, % (n)	26.0% (252) [0.0–61.4%]	9.7% (30) [1.2–18.7%]	22.0% (282)
Separate drinking water, % (n)	32.0% (311) [0.0–65.8%]	57.7% (179) [45.7–74.7%]	38.3% (490)
Spent ≥2 days without water in last 30 days, % (n)	44.1% (428) [15.7–75.4%]	33.2% (103) [23.5–46.3%]	41.5% (531)
Did not have enough water in past 30 days, % (n)	37.3% (362) [7.6–71.1%]	23.9% (74) [13.3–37.8%]	34.0% (436)
Modified hygiene due to lack of water, % (n)	28.2% (274) [7.6–68.4%]	19.4% (60) [13.3–28.1%]	26.1% (334)
Percent of income used to buy water, mean (SD)	20.8% (13.9) [14.0–42.4%]	19.7% (10.4) [18.2–21.0%]	20.5% (13.1)
Consumption lpcd, median (lower, upper quintile)	71.4 (47.6–95.2)	53.6 (35.7–71.4)	63.5 (42.9–95.2)
Mean FCR mg/Liter (lower, upper quintile), n	0.04 (0.0, 0.1), 937	0.01 (0.0, 0.0), 310	0.03 (0.0, 0.0) 1207
HH with > 0 mg/L FCR, %(n)	24.4% (237) [1.2–100%]	7.4% (23) [0.0–22.2%]	20.3% (260)
HH with > 0.1 mg/L FCR %(n)	4.9% (48) [0.0–45.3%]	1.3% (4) [0.0–4.2%]	4.1% (52)
Mean pH (lower, upper quintile), n	7.9 (7.8, 8.0) 968	7.9 (7.8, 8.0) 310	7.9 (7.8, 8.0) 1238
Access to clean/functional toilet, % (n)	96.2% (934) [91.1–100%]	91.6% (284) [74.4–98.7]	95.1% (1218)
Observed: Clean/functional toilet, % (n)	67.5% (655) [22.4–85.4%]	52.3% (162) [30.5–62.5%]	63.8% (817)
Mean (SD) users per toilet	7.4 (4.1) [6.0–8.8]	8.2 (6.3) [7.1–11.0]	7.6 (4.7)
HHs use shared toilet, % (n)	15.9% (220) [1.5–30.8%]	34.5% (107) [21.0–52.4%]	25.5% (327)
HHs could not find/afford hygiene items, % (n)	74.0% (719) [54.2–97.4%]	78.7% (244) [76.9–95.1%]	75.2% (963)
Observed: Soap and water at handwash station % (n)	50.8% (493) [7.9–67.1%]	45.5% (141) [22.0%–59.3%]	49.5% (634)
Observed: Soap or water at handwash station % (n)	80.1% (778) [47.4–93.9%]	69.0% (214) [57.3–76.5%]	77.4% (992)
HH left garbage in open, % (n)	1.6% (16) [0.0–10.1%]	11.9% (37) [6.7–20.7%]	4.1% (53)
HH under WSP program % (n)	46.7% (453) [0.0–100%]	0% (0) [0.0–0.0%]	35.4% (453)
HH reported at least one child < 5 years had diarrhea in past 2 weeks %, (n)	31.6% (307) [10.5–47.2%]	36.5% (113) [28.4–41.5%]	32.8% (420)

Abbreviations: household (*HH*), standard deviation (*SD*), free chlorine residual (*FCR*)

#### Water supply

**Fig. 2:**
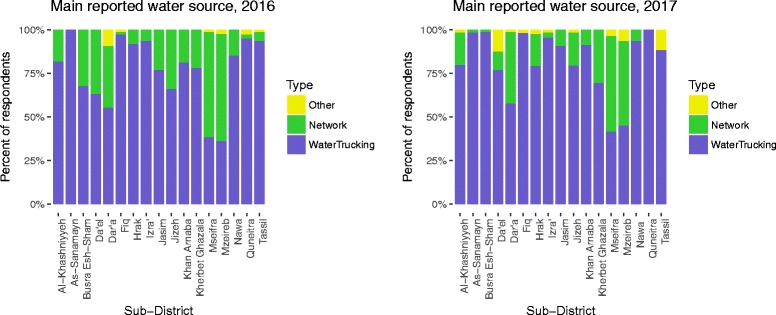
Main reported drinking water source in past 30 days by sub-districts

**Fig. 3:**
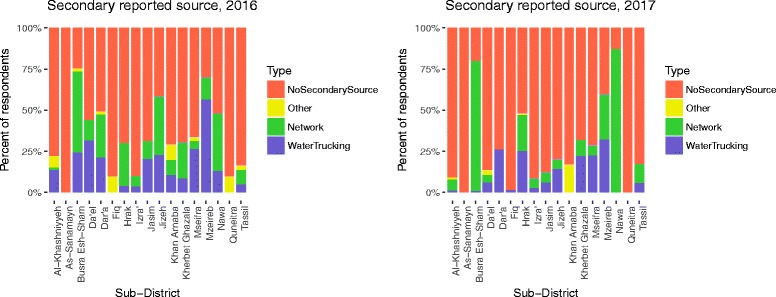
Secondary reported water source in past 30 days by sub-districts

**Fig. 4:**
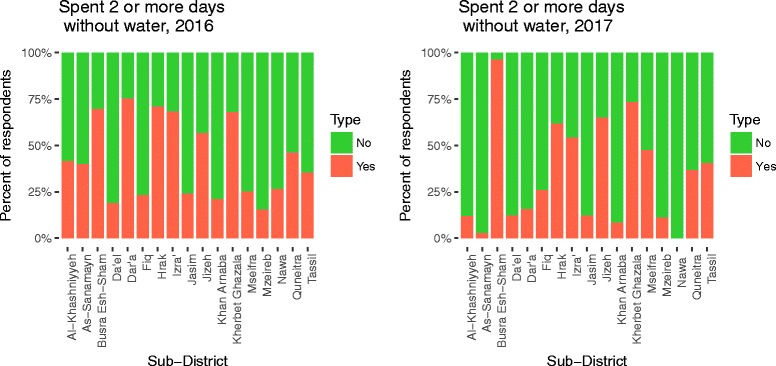
Spent 2 or more days without water in past 30 days by sub-districts

**Fig. 5:**
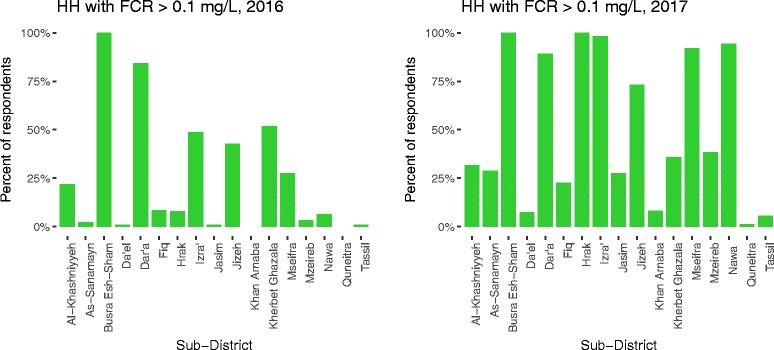
Households with FCR > 0.1 mg/L by sub-districts

Water trucking was reported as the “most used source in last 30 days” by 77.0% of total respondents, ranging from 36.1–100% by sub-district (Table 1, Fig. 2). Piped water supply on the premises, referred as “network” hereafter, was reported as the most used source of water by 22.0% of respondents, with range by sub-district of 0–61.4%. Overall, 32.2% reported a secondary source of water (Fig. 3). Additionally, 38.3% of respondents reported separating their drinking water from other domestic uses, 41.5% of respondents reported spending two or more days without water (Fig. 4), 34.0% of respondents reported they did not have enough water sometime in the last 30 days, and 26.1% of respondents reported they modified their hygiene practices to adjust for lack of water, an indicator of their own perception of their water security. Respondents reported paying, on average, 20.5% of their income for water. Self-reported median water consumption was 65.3 l/capita/day (lpcd). Mean FCR was 0.03 mg/L, with the majority of household water (79.7%) having 0.00 mg/L FCR, and 4.1% having > 0.1 mg/L (Fig. 5).

#### Sanitation and hygiene

**Fig. 6:**
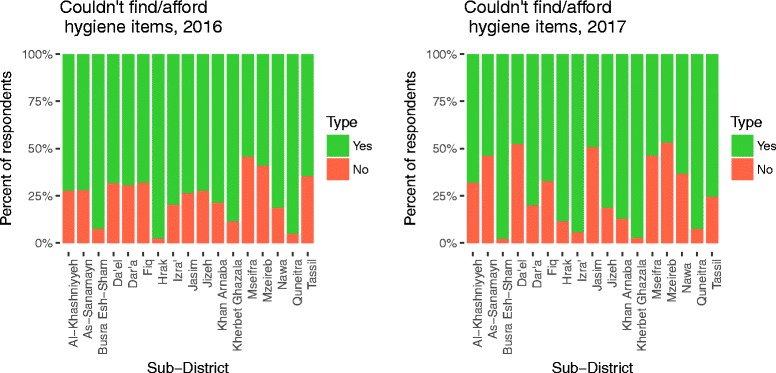
Percentage of respondents who could not find or afford hygiene items in past 30 days by sub-districts

Overall, 95.1% of respondents reported having access to a clean and functional toilet for household members, ranging from 74.4–100% by sub-district; 63.8% of respondents were able to show that toilet to the enumerators (Table 1). The average number of users per toilet was 7.6 persons, mostly family members; and 25.5% reported using a shared toilet. During the survey, 49.5% of respondents showed a handwashing station with both soap and water to the enumerator; 77.4% showed a handwashing station with either soap or water. Overall, 4.1% of households reported leaving their garbage in the open, and 35.4% were in communities targeted with Water Safety Plan programming. When asked a non-specific open question about hygiene access, 75.2% of the sample population reported not being able to find or afford necessary hygiene items in the market (Fig. 6). The respondents who answered they could not find or afford items were asked in follow-up what items they could not find or afford, respondents self-reported the following items: washing powder (59.3%), shampoo (58.2%), dish detergent (56.1%), house cleaners (49.7%), bar soap (29.2%), disposable diapers (53.6%), sanitary pads (41.3%), garbage bags (19.0%), jerrican/bucket (17.8%), towel (16.2%), toothpaste (7.6%), washing line (5.2%), toothbrush (4.6%), comb (3.4%), and nail clippers (3.0%). When asked another follow-up about individual items, > 95% of respondents reported affordability as the reason for not having the item, except for garbage bags, where it was > 80%.

#### Health

Lastly, 32.8% of households reported at least one child < 5 had diarrhea in the last two weeks (Table 1).

### 2017 Household WASH assessment

In the second survey round, conducted with the same population using a same questionnaire in February 2017, the majority of indicators were statistically significantly different (Table 2). Demographically, there were fewer people per household (7.1), a lower percentage of IDP households (24.5%), and a lower mean age of respondent (40.9 years).

**Table 2:** Descriptive statistics from 2017 survey results and comparison with 2016 result

	Governorate [min and max per sub district]		Total	*p*-value*
	Dar’a	Quneitra		
HH has at least one child < 5 years, % (n)	71.4% (1112) [59.0–90.5%]	67.9% (248) [63.5–72.1%]	70.8% (1360)	0.703
Mean (SD) people per household	7.2 (3.2) [6.0–8.5]	6.9 (3.4) [5.4–8.8]	7.1 (3.3)	0.001
IDP households, % (n)	21.8% (242) [4.6–52.6%]	37.0% (91) [9.8–81.5%]	24.5% (333)	< 0.001
Female headed HH, % (n)	17.1% (190) [0.0–51.6%]	33.5% (88) [16.0–53.8%]	20.1% (273)	0.682
Mean (SD) age of the head of HH	41.1 (11.1) [37.3–46.0]	40.1 (10.2) [36.6–43.8]	40.9 (11.0)	0.002
Families in damaged/shared shelters, % (n)	49.4% (549) [15.3–94.1%]	31.5% (78) [11.5–50.8%]	46.1% (627)	0.828
Water trucking main source in last 30 days, % (n)	80.4% (894) [41.7–100%]	91.9% (228) [80.0–100%]	82.5% (1122)	< 0.001
Network main source in last 30 days, % (n)	17.1% (190) [0.0–54.8%]	7.3% (18) [0.0–18.7%]	15.3% (208)	< 0.001
Separate drinking water, % (n)	12.8% (142) [0.0–68.2%]	9.7% (24) [0.0–21.3%]	12.2% (166)	< 0.001
Spent ≥2 days without water in last 30 days, % (n)	39.1% (435) [0.0–96.5%]	21.4% (53) [8.5–36.9%]	35.9% (488)	0.003
Did not have enough water in past 30 days, % (n)	29.3% (326) [0.0–80.0%]	6.9% (17) [4.6–8.5%]	25.2% (343)	< 0.001
Modified hygiene due to lack of water, % (n)*	15.2% (169) [0.0–48.2%]	3.6% (9) [1.3–4.9%]	13.1% (178)	< 0.001
Percent of income used to buy water, mean (SD)	22.0% (15.3) [11.7–38.6%]	21.4% (14.7) [14.8–28.9%]	21.9% (15.4)	< 0.001
Consumption lpcd, median (lower, upper quintile)	48.6 (35.7–71.4)	63.5 (47.6–95.2)	61.2 (42.9–86.3)	0.294
Mean FCR mg/Liter (lower, upper quintile), n	0.16 (0.0, 0.3), 1096	0.07 (0.0, 0.0), 248	0.15 (0.0, 0.3) 1344	< 0.001
HH with > 0 mg/L FCR, % (n)	56.7% (630) [0.0–100%]	17.3% (43) [8.5–32.0%]	49.5% (673)	< 0.001
HH with > 0.1 mg/L FCR % (n)	32.3% (359) [0.0–83.3%]	8.5% (21) [0.0–19.7%]	27.9% (380)	< 0.001
Mean pH (lower, upper quintile), n	7.9 (7.8, 8.0) 1093	7.8 (7.8, 8.0) 248	7.9 (7.8, 8.0) 1341	< 0.001
Access to clean/functional toilet, % (n)	96.2% (1070) [77.0–100%]	99.2% (246) [97.3–100%]	96.8% (1316)	0.029
Observed: Clean/functional toilet, % (n)	69.6% (774) [25.3–98.8%]	69.0% (171) [55.4–83.0%]	69.5% (945)	0.002
Mean (SD) users per toilet	7.1 (3.6) [6.0–8.4]	6.5 (3.7) [5.1–8.7]	7.0 (3.6)	0.001
HHs use shared toilet, % (n)	12.7% (141) [1.0–32.8%]	11.3% (28) [6.4–16.4%]	12.4% (169)	< 0.001
HHs could not find/afford hygiene items, % (n)	73.7% (819) [46.8–97.7%]	77.8% (193) [67.2–92.3%]	74.4% (1012)	0.651
Observed: Soap and water at handwash station % (n)	49.6% (551) [7.4–82.1%]	44.4% (110) [12.8%–69.3%]	48.6% (661)	0.648
Observed: Soap or water at handwash station % (n)	77.2% (859) [44.3–97.6%]	72.6% (180) [62.3–82.7%]	76.4%(1039)	0.525
HH left garbage in open, % (n)	1.5% (17) [0.0–7.7%]	7.3% (18) [3.3–12.0%]	2.6% (35)	0.026
HH under WSP program % (n)	50.5% (562) [0.0–100%]	0.0% (0) [0.0–100%]	41.3% (562)	0.002
HH reported at least one child < 5 years had diarrhea in past 2 weeks %, (n)	21.0% (233) [7.1–55.7%]	18.1% (45) [12.8–23.0%]	20.4% (278)	< 0.001

*The *p*-value is from two-sample *t*-test comparing 2016 and 2017 results

Abbreviations: household (*HH*), standard deviation (*SD*), free chlorine residual (*FCR*)

#### Water supply

A higher proportion of respondents reported using trucked water as the main source in the last 30 days, and fewer respondents reported separating drinking water from other domestic uses (12.2%), having two or more days without water in the last 30 days (35.9%), not having enough water in the past 30 days (25.2%), and modifying their hygiene practices due to lack of water (13.1%). The percent of income spent on water was slightly higher, at 21.9%. Consumption remained at 61.2 lpcd. However, FCR was higher, with 27.9% of household water having > 0.1 mg/L FCR.

#### Sanitation and hygiene

Reported and observed access to clean/functional toilets increased (96.8 and 69.5%, respectively), and mean users per toilet (7.0) and proportion sharing a toilet (12.4%) decreased. Handwashing indicators were not significantly different, and the proportion of households with WSP programming in their community increased to 41.3%.

#### Health

Lastly, past two weeks reported diarrhea rates in children were lower, at 20.4%.

### Univariate, multivariate, and mixed effect regression

Fifteen variables were created for inclusion in regression analysis, to assess the effectiveness of the WASH interventions (Table 3).

**Table 3:** Description of variables included in regression analysis

Code	Variable	Type
MainSource	The main reported source of water in past 30 days	Binary (piped network 1; trucked water 0)
MixedWater	If a household reported both a primary and secondary water source	Binary (mixed 1, one source only 2)
WaterUse	Amount of water consumed by the household	Continuous (lpcd)
AdequateWater	If a household had adequate water as defined by self-reported sufficient water and not being out of water more than two days	Binary
IncomeSpent	The percent of income spent on water	Continuous (%)
SeparateWater	If the household respondent reported separating drinking and non-drinking water	Binary
FCR	FCR in household water	Continuous (in mg/L)
FCR_Bin	FCR as binary variable with measured values > 0.1 mg/L	Binary
FunctionalToilet	If the household respondent self-reported they had access to a functional and clean non-shared toilet facility with less than 10 users	Binary
Handwashing	If soap and water were present in a handwashing station in the home	Binary
HygieneAccess	If the household respondent reported they could not find and afford necessary hygiene items	Binary
WasteDisposal	If the household respondent reported waste was collected regularly at least once a month and disposed of in a designated site	Binary
Displacement	If the household was internally displaced	Binary
Shelter	If the household shelter was damaged	Binary
WSP	If the household was in a WSP community	Binary

Abbreviations: free chlorine residual (*FCR*), water safety plan (*WSP*)

In 2016, 10 of the 15 variables were significantly associated with diarrhea in children < 5 in univariable analysis (Table 4). Protective factors included HygieneAccess (unadjusted odds ratio (uOR): 0.58 (95% confidence interval (CI): 0.53–0.63)); FunctionalToilet (0.56, 0.52–0.60); WasteDisposal (0.74, 0.68–0.82); Handwashing (0.68, 0.63–0.74); and, WSP (0.68, 0.62–0.74). Risk factors included AdequateWater (2.00, 1.56–2.56); SeparateWater (2.63, 1.91–3.64); Displacement (1.58, 1.29–1.92); and, Shelter (1.40, 1.18–1.65). IncomeSpent was significantly associated (1.03, 1.02–1.04), with diarrhea in children < 5. MainWater, MixedWater, WaterUse, FCR, and FCR_Bin were not significant. In multivariate regression, four variables remained significant. The protective factor was FunctionalToilet (adjusted OR (aOR): 0.62 (95% CI 0.46–0.82)). Risk factors included AdequateWater (2.14, 1.62–2.84) and SeparateWater (2.03, 1.52–2.72). IncomeSpent was significant, with OR near one (1.03, 1.02–1.04). These four variables remained significant in mixed effects regression. The protective factor was FunctionalToilet (mixed effect OR (mOR): 0.56 (95% CI 0.43–0.72)). Risk factors included AdequateWater (2.11, 1.61–2.76)) and SeparateWater (2.04, 1.55–2.70). IncomeSpent was significant with OR near one (1.03, 1.02–1.04).

**Table 4:** Univariate, multivariate, and mixed effect regressions of 2016 dataset using reported diarrhea outcome of children < 5 years old over two weeks period as dependent variable

Variables^a^	Unadjusted	Adjusted	Mixed effect
	OR (95% CI)^b^	OR (95% CI)^b^	OR (95% CI)^b^
Reported separating drinking and non-drinking water (SeparateWater)	2.63 (1.91–3.64)	2.03 (1.52–2.72)*	2.11 (1.61–2.76)*
Reported access to toilet (FunctionalToilet)	0.56 (0.52–0.60)	0.62 (0.46–0.82)*	0.56 (0.43–0.72)*
Reported access to sufficient water when needed (AdequateWater)	2.00 (1.56–2.56)	2.14 (1.62–2.84)*	2.04 (1.55–2.70)*
The percent of income spent on water (IncomeSpent)	1.03 (1.02–1.04)	1.03 (1.02–1.04)*	1.03 (1.02–1.04)*
Observed soap and water at handwashing station (Handwashing)	0.68 (0.63–0.74)	1.04 (0.77–1.40)	
Reported access to hygiene items (HygieneAccess)	0.58 (0.53–0.63)	0.82 (0.58–1.15)	
Reported status of the shelter (Shelter)	1.4 0(1.18–1.65)	1.08 (0.82–1.43)	
Reported status of internal displacement (Displacement)	1.58 (1.29–1.92)	1.26 (0.95–1.67)	
Reported access to waste disposal (WasteDisposal)	0.74 (0.68–0.82)	0.82 (0.62–1.09)	
Household included in WSP (WSP)	0.68 (0.62–0.74)	1.03 (0.75–1.40)	
Reported main water source (MainSource)	1.02 (0.88–1.18)		
Reported access to secondary water source (MixedWater)	1.03 (0.90–1.18)		
Household reported water consumption (WaterUse)	1.0 (0.998–1.001)		
FCR as binary variable (FCR_Bin)	1.08 (0.78–1.48)		
Measured FCR (FCR)	1.14 (0.34–3.43)		

^a^Consult Table 3 for detailed description of the variables

^b^Odds ratio and 95% confidence interval

*statistically significant association (*p*-value < 0.05)

In 2017, 10 of the 15 variables were significantly associated with diarrhea in children < 5 years in univariable analysis (Table 5). Protective factors included HygieneAccess (uOR: 0.58 (95% CI: 0.52–0.64)); FunctionalToilet (0.70, 0.64–0.77); SeparateWater (0.80, 0.68–0.96); FCR (0.20, 0.08–0.44); FCR_Bin (0.54, 0.49–0.59); WSP (0.74, 0.67–0.82); Handwashing (0.61, 0.56–0.66); MainWater (0.71, 0.61–0.82); and, MixedWater (0.77, 0.68–0.86). The risk factor was Shelter (1.20, 1.02–1.40). WaterUse, WasteDisposal, Displacement, IncomeSpent, and AdequateWater were not significant. In multivariate regression, two variables remained significant, with HygieneAccess (aOR: 0.58 (95% CI 0.40–0.84) and Handwashing (0.73, 0.54–0.99) protective. Both variables remained significant in mixed effects regression; with HygieneAccess (mOR: 0.55 (95% CI 0.39–0.79)) and Handwashing (0.66, 0.48–0.90) protective (Table 5).

**Table 5:** Univariate, multivariate, and mixed effect regressions of 2017 dataset using reported diarrhea outcome over two weeks period as dependent variable

Variables^a^	Unadjusted	Adjusted	Mixed effect
	OR (95% CI)^b^	OR (95% CI)^b^	OR (95% CI)^b^
Reported access to hygiene items (HygieneAccess)	0.58 (0.52–0.64)	0.58 (0.4–0.84)*	0.55 (0.39–0.79)*
Observed soap and water at handwashing station (Handwashing)	0.61 (0.56–0.66)	0.73 (0.54–0.99)*	0.66 (0.48–0.90)*
Reported separating drinking and non-drinking water (SeparateWater)	0.80 (0.68–0.96)	0.91 (0.57–1.42)	
Reported access to toilet (FunctionalToilet)	0.70 (0.64–0.77)	0.84 (0.62–1.13)	
Reported main water source (MainSource)	0.71 (0.61–0.82)	0.92 (0.58–1.42)	
Measured FCR (FCR)	0.20 (0.08–0.44)	0.37 (0.04–1.38)	
FCR as binary variable (FCR_Bin)	0.54 (0.49–0.59)	0.91 (0.47–2.13)	
Reported status of the shelter (Shelter)	1.20 (1.02–1.40)	1.14 (0.85–1.53)	
Household included in WSP (WSP)	0.74 (0.67–0.82)	0.82 (0.58–1.17)	
Reported access to secondary water source (MixedWater)	0.77 (0.68–0.86)	0.9 (0.62–1.31)	
Reported status of internal displacement (Displacement)	1.00 (0.85–1.17)		
Reported access to waste disposal (WasteDisposal)	0.91 (0.79–1.05)		
Household reported water consumption (WaterUse)	1.0 (0.999–1.002)		
The percent of income spent on water (IncomeSpent)	1.0 (0.999–1.00)		
Reported access to sufficient water when needed (AdequateWater)	1.09 (0.94–1.28)		

^a^Consult Table 3 for detailed description of the variables

^b^Odds ratio and 95% confidence interval

*statistically significant association (*p*-value < 0.05)

Regressions for 2016 data with outcome variable FCR are not presented due to low FCR concentrations. In 2017, for univariable analysis, 10 of the 13 non-FCR variables were significantly associated with FCR_Bin (FCR > 0.1 mg/L) (Table 6). Protective factors included FunctionalToilet (uOR: 1.16 (95% CI: 1.01–1.34)); MainWater (1.59, 1.23–2.05); MixedWater (3.95, 2.35–6.64); Handwashing (1.64, 1.34–2.00); SeparateWater (1.30, 1.03–1.64); HygieneAccess (0.72, 0.65–0.80); WasteDisposal (1.78, 1.36–2.34); and, WSP (6.70, 2.70–16.57). Risk factors included AdequateWater (0.54, 0.51–0.58) and Shelter (1.67 (1.36–2.04). WaterUse, Displacement, and IncomeSpent were not significant. In multivariate regression, eight variables remained significant. Protective factors included MainWater (aOR: 0.47 (95% CI 0.31–0.72); MixedWater (4.02, 2.89–5.63); AdequateWater (1.43, 1.03–2.01); Handwashing (2.36, 1.73–3.24); WasteDisposal (1.79, 1.25–2.60), and WSP (9.75, 6.85–14.05). Risk factors were SeparateWater (0.41, 0.25–0.65) and Shelter (1.36, 1.00–1.86). Four of these protective variables remained significant in mixed effect analysis: MainWater (mOR: 0.56 (95% CI 0.34–0.92); MixedWater (2.11, 1.34–3.32); Handwashing (1.80, 1.26–2.57); and, WSP (24.16, 5.93–98.5).

**Table 6:** Univariate, multivariate, and mixed effect regressions of 2017 dataset using free chlorine residual as dependent variable

Variables^a^	Unadjusted	Adjusted	Mixed effect
	OR (95% CI)^b^	OR (95% CI)^b^	OR (95% CI)^b^
Reported access to secondary water source (MixedWater)	3.95 (2.35–6.64)	4.02 (2.89–5.63)*	2.11 (1.34–3.32)*
Reported main water source (MainSource)	1.59 (1.23–2.05)	0.47 (0.31–0.72)*	0.56 (0.34–0.92)*
Household included in WSP (WSP)	6.7 (2.71–16.57)	9.75 (6.85–14.05)*	24.16 (5.93–98.48)*
Observed soap and water at handwashing station (Handwashing)	1.64 (1.34–2.00)	2.36 (1.73–3.24)*	1.80 (1.26–2.57)*
Reported separating drinking and non-drinking water (SeparateWater)	1.30 (1.03–1.64)	0.41 (0.25–0.65)*	0.94 (0.55–1.61)
Reported access to sufficient water when needed (AdequateWater)	0.54 (0.51–0.58)	1.43 (1.03–2.01)*	1.10 (0.71–1.72)
Reported status of the shelter (Shelter)	1.67 (1.36–2.04)	1.36 (1.00–1.86)*	1.12 (0.76–1.64)
Reported access to waste disposal (WasteDisposal)	1.78 (1.36–2.34)	1.79 (1.25–2.60)*	1.31 (0.85–2.01)
Reported access to toilet (FunctionalToilet)	1.16 (1.01–1.34)	1.07 (0.78–1.46)	
Reported access to hygiene items (HygieneAccess)	0.72 (0.65–0.80)	0.86 (0.60–1.23)	
The percent of income spent on water (IncomeSpent)	1.0 (0.997–1.01)		
Reported status of internal displacement (Displacement)	0.92 (0.81–1.05)		
Household reported water consumption (WaterUse)	1.0 (0.997–1.001)		

^a^Consult Table 3 for detailed description of the variables

^b^Odds ratio and 95% confidence interval

*statistically significant association (*p*-value < 0.05)

## Discussion

In opposition controlled Southern Syria, surveys conducted in 2016 and 2017 found that: 1) pipped water supply as main water source in the last 30 days declined from > 90% before the conflict to 22.0% in 2016 and 15.3% in 2017, with privately operated water trucking networks filling the gap; 2) water security was a moderate concern, as households accessed 50–60 lpcd, but some households reported days without water, not having enough water, and modifying hygiene practices due to lack of water; 3) water safety improved from 2016 to 2017, as households with > 0.1 mg/L FCR increased from 4.1% to 27.9%, with households in communities targeted with WSP programming 24.16 times more likely to have FCR > 0.1 mg/L; additionally households reporting trucked water as the main source were more likely to have FCR > 0.1 mg/L (as trucked water can be chlorinated at collection); 4) the majority of households had access to a clean and functional toilet, a handwashing station with soap and/or water, and a garbage disposal method; 5) the proportion of households where respondents self-reported childhood diarrhea over a period of two weeks declined from a high (but commonly seen in emergency situations) of 32.8% to a more moderate level of 20.4% from 2016 to 2017; and, 6) sanitation (functional toilet) and hygiene (hygiene access and reported handwashing) indicators were protective against diarrheal disease in children in 2016 and 2017, respectively; some water supply indicators were identified as risk factors; these results should be interpreted with caution as further research on household water practices is needed to contextualize these results.

While this data highlights promising trendlines between 2016 and 2017, this water access comes at high cost, as respondents consistently reported paying ~ 20% of their income on water. While markets remain functional, and hygiene items were available, respondents also reported they could not afford consumable hygiene items such as washing powder, shampoo, dish and household cleaners, menstrual hygiene products, and diapers. Overall, the results indicate the private sector has effectively replaced decaying piped water infrastructure, with services that would be considered “basic” under the new Sustainable Development Goals [19]. However, these services absorb a high percentage of household disposable income, and as household purchasing power is low, water safety remains an issue. Despite all this, WSP programming successfully reduced the risk of disease transmission by increasing FCR concentrations.

The ideal WASH solution in Syria would be to maintain and restore pre-existing infrastructure services to a level considered “safely managed” under the Sustainable Development Goals. However, infrastructure requires support systems; all of these have been strained by protracted conflict. The estimated operations and maintenance costs for this infrastructure greatly exceeds humanitarian funding levels available for WASH response in Syria. The data herein thus raises questions on: 1) How should responders support affected populations in accessing safe, sufficient, and reliable drinking water supplies at reasonable cost? 2) Should hygiene kits be distributed in kind in contexts where hygiene products are widely available in markets? 3) How can community-level WASH interventions, such as WSPs, be scaled up? and, 4) What are the appropriate criteria to use to prioritize beneficiaries when there are insufficient resources and capacities, respecting the humanitarian principles of equity and universality?

Recently, there has been international focus on market-based solutions in emergencies, including providing support and regulation to markets and providing direct cash transfers to affected populations [20, 21]. These solutions are particularly appropriate for emergencies in urban settings, where the private sector can become the main WASH service provider. When compared to current standard practice (distribution of pre-packaged hygiene kits that may not meet beneficiary needs), using markets has the positive aspect that affected populations can obtain what they need sustainably [10]. Provided the service is adequate, in unregulated environments there are two negative consequences for users: affordability and quality. Affordability can be addressed with well-targeted subsidies, including coupons or vouchers [20, 21]. However, quality of water delivered by unregulated vendors remains a concern.

Water Safety Plans (WSPs) are a comprehensive risk assessment and management approach to water delivery, with the goal of ensuring drinking water safety by preventing or minimizing contamination. [22] The five steps in developing a WSP are: preparation; system assessment; monitoring; management and communication; and, feedback and improvement. WSPs are generally implemented in large utilities in development contexts, and have not traditionally been considered an emergency response activity. In southern Syria, the WSP approach targeted the middle (public and private wells, piped network, chlorination stations) and end of the water chain (consumers), including vendors that transport water and users that store it. Key lessons for success were: 1) extensive mobilization and follow-up at the household level using a volunteer network; 2) focusing on the key, simple parameter of FCR with pooltesters for operational monitoring and verification; and, 3) flexibility in adapting the programmatic strategy with evidence. The Syrian experience shows the WSP approach can yield results in emergencies. Community-level WSP programming also reaches a scale that allows cost-effective use of limited resources to reach those most in need.

The limitations of this analysis include: 1) southern Syria is not representative of all Syria, especially for power supply, and therefore water network, availability; 2) no microbiological water quality data was collected, although FCR presence is an indicator of no/low bacterial contamination; 3) self-reported diarrhea is an indicator subject to response bias, and no standard definition of diarrhea was provided to respondents; 4) sample size calculation was not specifically completed for households with children < 5 and we removed a large number of households from the dataset; 5) data are from cross-sectional surveys, not experimental evaluations; thus, causation cannot be determined; and, 6) the survey was not designed to distinguish between hygiene item availability and affordability, which are different constructs, and further research is necessary to disaggregate the reasons for lack of hygiene items. Results should be understood within these limitations.

The results presented herein highlight the importance of collecting data locally in emergencies to develop appropriate, targeted response activities to ensure water and sanitation access and reduce the risk of disease transmission. The Syrian experience presents a new and unique challenge by combining protracted conflict with a middle-income context with pre-existing infrastructures, and questions the traditional WASH humanitarian response. Markets, the private sector, and water vendors should not be overlooked: in contexts with a market capable of satisfactory WASH service provision, markets-based programming can be leveraged to reach larger populations and to build and encourage resilience. Additionally, authorities should be seen not only as service providers, but regulators. The risk management approaches in WSPs should not be considered only a development intervention, but instead can be considered a primary emergency response intervention to ensure water safety. The lesson from the Syria WASH response is that allowing market forces to manage services and quantity, and targeting response activities on increasing affordability, quality, and regulation can be an effective, scalable, and cost-effective strategy to guarantee the human right to water and sanitation in protracted emergencies.

## Conclusions

Syria is an example of an emerging complex emergency. Based on about 2000 surveys conducted in opposition controlled southern Syria, we found that piped water supply on the premises as the main water source in the last 30 days declined significantly both in 2016 and in 2017, with privately operated water trucking networks filling the gap. Additionally, water safety improved from 2016 to 2017, with communities targeted with WSP programming were more likely to have FCR in households stored water. Lastly, the data showed that sanitation and hygiene indicators were protective against diarrheal disease in children. The lesson from the Syria WASH response is that allowing market forces to manage services and quantity, and targeting response activities on increasing affordability, quality, and regulation can be an effective, scalable, and cost-effective strategy to guarantee the human right to water and sanitation in this new type of protracted complex emergency settings with prior infrastructure and vibrant markets.

## Abbreviation

FCR: Free Chlorine Residual
HH: Household
HNO: Humanitarian Needs Overview
IDP: Internally Displaced People
LPCD: Liters per capita per day
OR: Odds Ratio
SD: Standard Deviation
WASH: Water, Sanitation, and Hygiene
WoS: Whole of Syria
WSP: Water Safety Plan
